# Nationwide Online Survey Enables the Reevaluation of the Safety of *Coleus forskohlii* Extract Intake Based on the Adverse Event Frequencies

**DOI:** 10.3390/nu11040866

**Published:** 2019-04-17

**Authors:** Chiharu Nishijima, Tsuyoshi Chiba, Yoko Sato, Keizo Umegaki

**Affiliations:** 1 Department of Food Function and Labeling, National Institute of Health and Nutrition, National Institutes of Biomedical Innovation, Health and Nutrition, 1-23-1 Toyama, Shinjuku-ku, Tokyo 162-8636, Japan; c-nishijima@nibiohn.go.jp (C.N.); tyschiba@nibiohn.go.jp (T.C.); satoyoko@nibiohn.go.jp (Y.S.); 2 Department of Food Safety and Management, Showa Women’s University, 1-7-57 Taishido, Setagaya-ku, Tokyo 154-8533, Japan

**Keywords:** dietary supplements, safe intake, diarrhea, gastrointestinal stress, effectiveness

## Abstract

The formulations of the functional ingredients of dietary supplements was studied with a small number of subjects, with a particular focus on their effectiveness, but not enough to evaluate their safety. In this regard, the reevaluation and estimation of the safe use of marketed products, with regards to their adverse event (AE) frequencies, are important. To address this issue, a post-marketing nationwide online survey was conducted for the herbal ingredient *Coleus forskohlii* extract (CFE), a popular weight-loss ingredient. The questionnaire included product names, adherence to the claimed amount, and AE experiences. The safe intake amount was estimated by the relationship between the claimed amount of CFE and the frequencies of AEs of each product. The number of users who experienced AEs was 75 (10.5% of all users). Gastrointestinal symptoms accounted for 92.0% (*n* = 69) of all AEs, and diarrhea alone accounted for 81.3% (*n* = 61). The amount of CFE was significantly associated with the occurrence of diarrhea (*p* = 0.005). The fitted curve showed that the safe intake amount of CFE was less than 250 mg/day; however, considering its effectiveness, 500 mg/day of CFE might be acceptable. In conclusion, nationwide online surveys of users enable us to confirm and reevaluate the safety of herbal supplements.

## 1. Introduction

The use of dietary supplements, especially those containing herbal ingredients, has increased in recent times [1]. Herbal ingredients used as dietary supplements are often traditionally used for health remedies or are consumed as a part of the diet and are recognized as safe. However, when used in their concentrated form, as tablets and capsules, their safety remains a concern [2]. Recent studies have revealed that herbal products, especially those used for weight loss, can cause adverse events (AEs), such as serious liver damage [1,2,3] and gastrointestinal stress [4,5].

Setting an appropriate intake amount of herbal ingredients affects both their effectiveness and safety. The intake amount of food ingredients is determined on the basis of animal studies. However, the pharmacological actions of chemicals often differ between species [6,7], and it is ideal to set the optimal amount of herbal ingredients, on the basis of human data. Randomized controlled trials (RCTs) are conducted as a means of premarketing evaluation, often with a few dozen people. RCTs can demonstrate the efficacies of dietary supplements but cannot sufficiently demonstrate their safety, because the frequency of AEs related to dietary supplements is partly induced by individual gender, age, genetics, and lifestyle. Thus, the frequency of AEs is as low, as approximately 4% of dietary supplement users, and thus, the few dozen people in RCTs, is not a large enough sample to fully understand AEs [4,8,9]. Moreover, it is considered that the current passive AE report collecting system is insufficient to detect the potential harm brought about by dietary supplements [10]. Thus, to ensure the safety of dietary supplements, post-marketing surveillance, directly and actively contacting consumers would be an effective measure [11].

In our previous study, we had showed that nationwide online surveys of dietary supplement users were useful in collecting a number of self-reported AEs that demonstrated the presence of suspected harmful ingredients involved in diarrhea and skin manifestations, due to dietary supplement use [5,12]. In the study regarding diarrhea [5], the use of CFE accounted for the majority (61–81%) of all herbal products that were claimed to have caused diarrhea; however, the result may have reflected the recent increase in sales. CFE has been used for centuries in Ayurvedic medicine to treat various diseases of the cardiovascular, respiratory, and central nervous systems [13]. It is a popular herbal weight-loss ingredient in dietary supplements in Japan and is standardized by the active diterpene compound forskolin. The safety of the standardized CFE has been tested by conducting RCTs with 250 mg to 1000 mg of CFE, with a resultant approximately 20% mild adverse gastrointestinal events, in the entire dose range [14]. However, animal studies showed liver damage at a dose level equivalent to almost 3000 mg/60 kg body weight for humans [15,16,17]. To date, little is known about the frequencies of AEs and the relationship between the amount and the occurrence of AEs, when a large population is exposed to CFE through marketed products.

In this study, we conducted a nationwide online survey to collect the experiences of AEs among CFE dietary supplement users. Using the data, we tried to figure out the dose–diarrhea frequencies and then estimate the safe intake amount of CFE ingredient. We also determined whether the onset of liver damage observed in the animal model [15,17] was also found in our survey.

## 2. Materials and Methods

### 2.1. Online Survey Procedure

A nationwide online survey, with previously validated results, was conducted by Rakuten Research, Incorporated (Tokyo, Japan) [5]. The research company is one of the companies created by the Rakuten Group, which hosts a variety of services, including an online shopping site and holds over 1.2 billion global members (as of February 2019), and the research registrants are composed of those member users. The company takes measures to prevent and eliminate fraudulent responses, by using computerized and visual quality check systems.

The survey was divided into the following two phases—a preliminary survey to screen the users, and a full-scale survey to collect detailed user information. In identifying and confirming the product name, we asked the users to enter the product and the manufacturer name twice, once in the preliminary survey and once in the full-scale survey; because the names of the supplementary products often reflect the names of their functional components or ingredients, it was easy to make a random guess when users were not sure of the exact name of the products they took. Then, only the users with absolute agreement were considered to be eligible for further analysis. A three-day washout was also inserted between the preliminary survey (conducted between 1 and 3 December 2017) and the full-scale survey (conducted between 7 and 11 December 2017). The surveys were conducted with the request of at least 1000 complete responses. This study was approved by the Research Ethics Committee of the National Institutes of Biomedical Innovation, Health and Nutrition (No. 221, approved on 27 October 2017) and was conducted in accordance with the Declaration of Helsinki.

### 2.2. Study Population and Questionnaire

**Figure 1:**
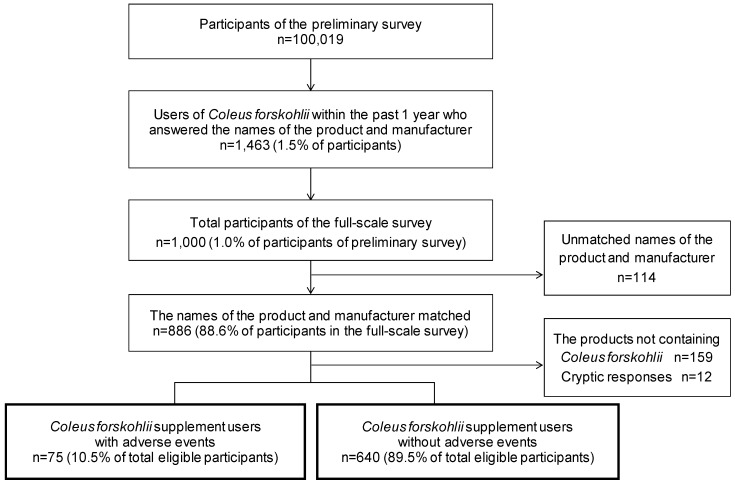
Flow diagram for the selection of study participants.

The research invitation was emailed to registrants who were 20 years or older, living across Japan. In the preliminary survey, users (*n* = 100,019) were asked about the experience of taking a dietary supplement containing CFE (yes/no) (Figure 1). Questions regarding the time of the experience and the names of the product and the manufacturer followed, if the answer to having an experience was “yes.” Subsequently, the preliminary users who answered “yes” regarding the intake of dietary supplements containing CFE in the previous year, and who had entered the name of the product and manufacturer (*n* = 1463) were invited for the full-scale survey. In the full-scale survey, the questionnaire included the name of the product and the manufacturer, and the experience of AEs (“no”/”yes” and multiple choices on 10 symptoms), the confidence level of the causal relationship between AEs and the CFE supplement use (“not sure,” “possible,” “probable,” or “highly probable”), and either one or multiple choices to the following question items—duration and frequency of supplement use, amount used per day, compared to the suggested amount on the label, use of the product after onset of adverse symptoms, health status when in use, and purpose of use. For symptoms, “worsening of liver function test” was set as an indicator for liver damage, due to self-assessed AE reports. A total of 1000 responses were delivered from the research company.

Among the product and manufacturer name responses we obtained in the preliminary and the full-scale surveys, the exactly matched responses (*n* = 886) were further confirmed at the manufacturers’ website, to check that they were existing products, and we obtained all the ingredient information from the website. A total of 715 users were left as eligible users after eliminating 159 users who indicated that the products did not contain CFE or who could not identify one definite product, as well as 12 cryptic users.

### 2.3. Analysis of the Survey Data

The analyses were performed on 715 users who identified the products. The dosage of CFE for the suggested servings per day, was obtained through the written information on either the manufacturer or retailers’ website, or telephone inquiries to the manufacturers. The experiences of AEs were classified, based on the self-assessed confidence level about the causal relationship [5]. The AEs that the respondents reported as having a causal relationship of “highly probable” and “probable” were classified as “users with experience of AEs,” and others were classified as “users without experience of AEs.” The frequencies of diarrhea (%) were calculated as “the number of diarrhea/total number of users” for each product.

Differences in distribution, compared according to experiences of AEs, were examined using the chi-squared or Fisher’s exact test and then further tested by performing residual analysis, when the chi-squared test was significantly different. The relationship between the intake amount of CFE and occurrence of diarrhea was analyzed through a logistic regression. Statistical analyses were performed using R version 3.4.3, and *p* values less than 0.05 were considered to be statistically significant.

## 3. Results

### 3.1. Characteristics

The participants—users of dietary supplements containing CFE—were mostly aged in their 30s to 50s in males, and 30s to 40s in females (Table 1). The number of users who self-reported an experience of AEs probably caused by CFE dietary supplements was 75 (10.5% of all participants, 9.5% of male, and 11.3% of female participants). The distributions of sex, age, and residential area of users were not different between those with and without experience of AEs. More users with experience of AEs reported CFE dietary supplement use within the past 1 month (users with experience of AEs, 36.0%; users without experience of AEs, 9.1%), whereas more users without experience of AEs continued their dietary supplement use over 1 year (users with experience of AEs, 17.3%; users without experience of AEs, 29.8%).

Most of the users took the supplements almost every day (77.8% in total) at the suggested amount (93.7%). The health status at the time they took the supplements was not significantly different between those with and without experience of AEs; 41.5% of all users took the supplements concomitantly with other dietary supplements, and 41.4% were in good health with no concerns about their health and took the supplement itself. Weight loss was the purpose of use for 86.6% of all users, with no difference between those with and without AEs, but maintenance of health was the purpose for more users without AEs (18.7% in those with AEs, 29.8% in those without AEs).

### 3.2. Adverse Symptoms

The most frequent symptoms that the users with AEs (*n* = 75) reported were gastrointestinal. Diarrhea, nausea and vomiting, and constipation accounted for 92.0% of all symptoms (*n* = 69); diarrhea alone made up 81.3% of all symptoms (*n* = 61) (Table 2). After the onset of the adverse symptoms, 55 users continued to take the dietary supplements without changing or reducing the supplement amount/frequency. Only 20 (26.7%) users reported that they stopped taking the supplements. One reported a worsening of liver function test but continued to take the supplements with reduced amount/frequency; there was no severe liver damage in the collected reports. In the further analysis, the stratification of symptoms, by sex, showed that the reports from female users were more centered on gastrointestinal stress (diarrhea, 88.6%; nausea and vomiting, 22.7%; headache, 6.8%; fatigue, 0%) than those from male users, which included more of the other symptoms (diarrhea, 71.0%; nausea and vomiting, 3.2%; headache, 19.4%; fatigue, 16.1%).

### 3.3. Relationship between the Suggested Amount of Coleus forskohlii Extract and Frequency of Diarrhea

**Figure 2:**
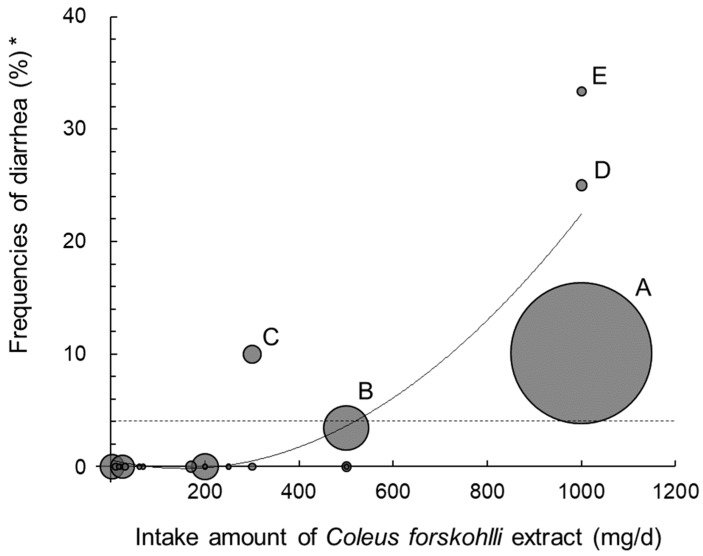
The relationship between the suggested amount of *Coleus forskohlii* extract per day and the frequencies of diarrhea (*n* = 61). Each circle represents a product pointed at the cross-point of the amount of *Coleus forskohlii* extract and the frequencies of diarrhea. The size of the circles indicates the number of the total users of each product. Six out of the 27 products were excluded due to the absence of information on the amount of the extract. The equation of the fitted curve is as follows: y = 3 × 10^−5^ × 2 − 0.0098x + 0.5862 (R^2^ = 0.7568). The dotted line shows the frequency of diarrhea caused by the dietary supplement use (4%) as a reference [5]. *Frequencies of diarrhea (%) were calculated for each product by “the number of diarrhea experiences/total users”; A, 56/557; B, 2/58; C, 1/10; D, 1/4; E, 1/3.

In total, 27 products containing CFE were identified. Five of them were reported to cause diarrhea when taken, which specified 250 mg/day to 1000 mg/day of CFE, as the suggested intake amount. The relationship between the suggested amount of CFE per day of each product and the frequencies of diarrhea is shown in Figure 2. Logistic analysis showed that the intake amount of CFE (mg/day) had a significant effect on whether the CFE users experienced diarrhea (*p* = 0.005). The products containing less than 250 mg/day of CFE (16 products) did not report any occurrence of diarrhea (69 users), whereas all three products containing 1000 mg/day of CFE (A, D, and E) had reported diarrheal experiences. Product A was the most popular product of all, with 557 users, and the occurrence of diarrhea was 56 (10.1%). The second most popular product, Product B, was used by 58 users, and the frequency of diarrhea was 3.4%. One report was collected from each user of Products C (number of users: 10, diarrhea: 10.0%), D (4, 25.0%), and E (3, 33.3%).

## 4. Discussion

In this survey, the relationship between the frequencies of diarrhea and the amount of CFE ingredient was determined. The data of the dose–diarrhea frequencies enabled us to suggest an acceptable intake amount of CFE, with consideration of its safety, as well as its effectiveness.

In the present survey, the CFE content of each product was assumed to be the same as the suggested amount on the product label. The amounts of the functional components of herbs in supplementary products often varied, despite their common origin and labeling [18]. However, marketed CFE has been standardized by forskolin, and we previously confirmed that the forskolin contents of marketed CFE products were consistent with the forskolin amount calculated by the CFE content written on the label, by analysis with a rapid HPLC, through the evaporative light scattering method [19]. Moreover, most of the users in this survey took the daily suggested amount. Thus, the suggested amount per day was presumed to reflect the intake amount. The products that the users took in our survey were those marketed in Japan, which contain CFE ingredients standardized at a 10% concentration of forskolin. Since CFE ingredients containing 20% of forskolin are also marketed in some other countries, an awareness of the standards is indispensable.

The AEs experienced by CFE supplement use were mostly diarrhea (81.3% of all AEs), and the diarrhea frequencies were 8.5% in all participants, which was higher than the previous report that focused on diarrhea symptoms, at approximately 4% of users [5]. In the report, approximately 60% used CFE supplements; thus, the frequency of 4% for diarrhea alone was higher than that generally seen [4,8,9]. There was no difference in the sex ratio of users with AEs, suggesting that there was no gender difference in the occurrence of AEs relating to CFE supplements. As with the female predominance in irritable bowel syndrome, women were more likely to suffer from gastrointestinal symptoms because ovarian hormones or the menstrual cycle can modulate intestinal function [20,21]. Women are also more likely to experience constipation and other somatic and visceral symptoms than men [20]. However, not all findings are consistent, and our result did not support the gender differences in symptoms. Moreover, 10.3% of participants were on a treatment for chronic disease when they used the CFE supplement. Although the specific disease condition was not asked in the questionnaire, health damages caused by the interaction of CFE with chronic disease, such as bleeding and hypotension, were not reported. There is a possibility that disease state or the drugs used may have independently caused diarrhea. The functional component of CFE, forskolin, has been shown to activate adenylate cyclase and increase the production of cAMP [22], to enhance lipolysis in fat cells [23,24] and in human studies [14,25,26]. However, it is suggested that the increased levels of cAMP in the intestine, caused by CFE, could lead to the secretion of water, which would manifest as diarrhea [14], and a similar mechanism of action has been observed for the cholera toxin [27]. Thus, it is assumed that diarrhea experienced by the CFE supplement users were caused by the CFE supplements.

The frequencies of diarrhea were shown to be CFE dose-dependent, which was consistent with the previous four-week dose escalation study [14]. In this previous study, adverse gastrointestinal events were observed, in approximately 20% of study subjects, in all the dose ranges between 250 mg/day and 1000 mg/day. The smallest amount of the occurrence of diarrhea in our study was 250 mg/day; thus, an amount less than 250 mg/day could be considered safe. According to the simulated equation of the fitted curve, the diarrhea frequency with a CFE amount of 500 mg/day, was lower than the diarrhea frequency of 4%, previously shown. Meanwhile, taking in 500 mg/day (250 mg/day CFE twice a day) of CFE has been reported to be effective for the management and treatment of obesity in overweight and obese men, according to an RCT which was previously conducted [25]. The users of all products that contained 1000 mg/day of CFE, reported diarrhea, with an accumulated percentage of 10.3%, which was higher than 4%. Although products containing CFE are popular as weight-loss supplements, diarrhea is not the intended mechanism for weight loss by CFE products. Accordingly, diarrhea occurring in 10.3% in products containing 1000 mg/day of CFE seemed to be too often. With regard to these facts, we estimated the safe level of CFE intake to be 250 mg/day; in addition, we suggest the maximum intake amount of 500 mg/day of CFE should be adopted as a reference amount for CFE product design, considering both its effectiveness and safety.

Although diarrhea is a relatively common gastrointestinal disorder, and is mostly minor and reversible, the liver damage sometimes reported by the use of herbal supplement can become fatal [3]. Animal testing is performed to determine the safe dose level for premarketing safety evaluation for food ingredients, and liver damage was observed by administering CFE in mice [15,17]. However, only one person in this study reported the worsening of liver function, with the self-assessed causal relation of “probable”. In this case, the participant continued taking the CFE supplement without going to a hospital, even after the worsening of liver function; thus, the case was considered not to indicate severe liver damage, and the liver damage detected in the mice studies was not found in this survey.

There are two possibilities for this difference regarding liver damage; one is species difference, and the other is the CFE dose. Our result of diarrhea caused by taking CFE for humans can be an example for species difference because diarrhea was not observed in mice, even in extreme amounts of CFE [15,17]. In contrast to diarrhea, liver damage might occur uniquely in mice and not in humans. Forskolin causes diarrhea by its activity in the intestine, indicating the non-involvement of absorption [14], whereas the constituents in CFE that induce hepatotoxicity, not including forskolin, are hypothesized to alter lipid metabolism and liver function [15,17]. Thus, we speculate the species differences in the absorption and the metabolism of the unidentified compound in CFE, brought about the difference in the liver damage effect between mice and humans. Meanwhile, a fatty liver in mice was induced with the administration of 0.5% CFE diets, which correspond to a dose of 600 mg CFE/kg body weight [17]; this equivalent CFE dose in humans is extrapolated to 49 mg/kg body weight [28]; i.e., 2940 mg/60 kg body weight. In the present survey, the maximum amount of CFE in the products used was designed to be 1000 mg/day. Since 97.5% of participants took CFE supplements at the suggested amount, it is unlikely that they consumed triple the suggested amount. Moreover, liver damage appeared by the first week of feeding, in mice, whereas most of our participants kept taking CFE supplements over a month, with no indication of liver damage. Therefore, it is reasonable to speculate that species difference is a large factor for the lack of observation of liver damage in the present study.

There are several limitations regarding the collection of information using a nationwide online survey. As the participants were registrants of an online research company who might be biased in age and socioeconomic background, they may not reflect CFE users in general. The results presented here, information related to CFE product use including AEs, were all self-reported by the users. The CFE contents of products were presumed from the label information because we confirmed the forskolin contents of the marketed products previously [19]; however, the products reported to be used were not separately analyzed in this survey. The severity and causal relation of the symptoms were not medically determined. Most AEs reported were gastrointestinal symptoms that develop acutely, and thus, CFE supplement users can recognize a causal relation. However, due to the survey nature of the study, proper causality analysis was not possible [29]; the involvements of other foods or food components that users took together with CFE dietary supplements or other confounders, such as diseases that cause diarrhea, were unknown, and thus, causality has not been established. Moreover, in contrast to diarrhea, liver damage progresses with no visible symptoms; thus, the participants cannot recognize this unless they take a physical checkup regularly, was not included in this survey. The findings about liver damage in this study need to be validated with further research taking a closer look at liver function. In addition, since herbal supplements are generally produced in multi-ingredient formulations, AEs may have been caused by ingredients other than CFE. Regarding diarrhea and liver damage, the symptoms were supported by the action of forskolin and other components in CFE, even though it is not clear which compound induces liver damage.

It is suggested that post-market monitoring should be considered as a tool to complement pre-market risk assessment for novel foods [11]. Likewise, in dietary supplements, the pre-market evaluation cannot fully demonstrate safety. In our study, a nationwide online survey enabled us to confirm the occurrence of AEs and present an acceptable intake amount, considering safety, by estimating from the dose–AE frequency. This was because data were collected from CFE users nationwide by conducting an online survey, which enabled us to have a large sample size. Compared to the results from RCTs, which are under one defined condition, data from the uses in miscellaneous conditions were obtained from people, who used the products with different CFE contents for various durations. Moreover, one of the key factors to re-estimating the amount of the ingredients is that the ingredient of CFE is standardized with forskolin. Herbal ingredients are composed of a mixture of numerous compounds that are mostly still unidentified. Chemical compositions within herbal ingredients vary, depending on the production areas, harvest time, and preparation methods of the manufacturers [30,31,32]; however, with standardized herbal extracts, such as CFE, we could assume the amounts of functional components in dietary supplements. Therefore, it is crucial to prepare herbs standardized by their functional components to obtain safety and efficacy data that are generalizable or transferable [33].

## 5. Conclusions

This study into the model of CFE revealed that a confirmation of the safety or reevaluation of the intake amount is possible, by using post-marketing surveillance using an online method. However, the estimation relies heavily on the quality of herbal extracts, and so it is crucial for the functional components to be standardized. Further studies are guaranteed to confirm the dose–AE relationship in other standardized ingredients.

## Figures and Tables

**Table 1:** Characteristics and use of *Coleus forskohlii* supplements according to those with/without experience of adverse events *.

		All		Users with Experience of Adverse Events (*n* = 75)		Users without Experience of Adverse Events (*n* = 640)		*p*
		*n*	(%)	*n*	(%)	*n*	(%)	
Sex	Male	326	(45.6)	31	(41.3)	295	(46.1)	0.434
	Female	389	(54.4)	44	(58.7)	345	(53.9)	
Age (Male)	20–29	10	(3.1)	2	(6.5)	8	(2.7)	0.574
	30–39	84	(25.8)	9	(29.0)	75	(25.4)	
	40–49	120	(36.8)	9	(29.0)	111	(37.6)	
	50–59	86	(26.4)	8	(25.8)	78	(26.4)	
	over 60	26	(8.0)	3	(9.7)	23	(7.8)	
(Female)	20–29	39	(10.0)	5	(11.4)	34	(9.9)	0.129
	30–39	120	(30.8)	21	(47.7)	99	(28.7)	
	40–49	144	(37.0)	12	(27.3)	132	(38.3)	
	50–59	64	(16.5)	5	(11.4)	59	(17.1)	
	over 60	22	(5.7)	1	(2.3)	21	(6.1)	
Residential area								
	Hokkaido	40	(5.6)	5	(6.7)	35	(5.5)	0.292
	Tohoku	41	(5.7)	8	(10.7)	33	(5.2)	
	Kanto	247	(34.5)	28	(37.3)	219	(34.2)	
	Chubu	116	(16.2)	10	(13.3)	106	(16.6)	
	Kinki	155	(21.7)	13	(17.3)	142	(22.2)	
	Chugoku, Shikoku	57	(8.0)	3	(4.0)	54	(8.4)	
	Kyushu, Okinawa	59	(8.3)	8	(10.7)	51	(8.0)	
Duration	<1 week	15	(2.1)	5	(6.7)	10	(1.6)	<0.001
	1 week–1 month	70	(9.8)	22	(29.3)	48	(7.5)	
	1–3 months	174	(24.3)	20	(26.7)	154	(24.1)	
	3–6 months	135	(18.9)	10	(13.3)	125	(19.5)	
	6 months–1 year	85	(11.9)	5	(6.7)	80	(12.5)	
	over 1 year	204	(28.5)	13	(17.3)	191	(29.8)	
	Did not remember	32	(4.5)	0		32	(5.0)	
Frequency	Almost every day	556	(77.8)	59	(78.7)	497	(77.7)	0.371
	Every other day	82	(11.5)	11	(14.7)	71	(11.1)	
	1–2 days/week	55	(7.7)	2	(2.7)	53	(8.3)	
	<1 day/week	17	(2.4)	2	(2.7)	15	(2.3)	
	Others	5	(0.7)	1	(1.3)	4	(0.6)	
Intake level compared with the amount suggested								
	Less	27	(3.8)	6	(8.0)	21	(3.3)	0.048
	Equal	670	(93.7)	69	(92.0)	601	(93.9)	
	More	18	(2.5)	0	(0.0)	18	(2.8)	
Health status (multiple answer)								
	In good health but had concerns about own health	106	(14.8)	16	(21.3)	90	(14.1)	0.094
	Having a treatment for chronic disease	74	(10.3)	9	(12.0)	65	(10.2)	0.620
	Taking a nonprescription drug	28	(3.9)	2	(2.7)	26	(4.1)	0.758
	Concomitantly with other health foods	297	(41.5)	27	(36.0)	270	(42.2)	0.304
	None of the above	296	(41.4)	29	(38.7)	267	(41.7)	0.612
Purpose of use (multiple answer)								
	Maintenance of health	205	(28.7)	14	(18.7)	191	(29.8)	0.043
	Weight loss	619	(86.6)	68	(90.7)	551	(86.1)	0.272
	Improvement of health	98	(13.7)	10	(13.3)	88	(13.8)	0.921
	Disease prevention	16	(2.2)	3	(4.0)	13	(2.0)	0.231
	Beauty care	49	(6.9)	2	(2.7)	47	(7.3)	0.152

* Statistical significance was tested between “users with experience of adverse events” and “users without experience of adverse events” using a chi-squared or Fisher’s exact test. Values in bold letters are significantly different from others within each question item, which was examined by performing residual analysis (*p* < 0.05).

**Table 2:** The prevalence of adverse events among all subjects (*n* = 715) and the use of the *Coleus forskohlii* product after the onset of adverse symptoms.

			Prevalence		Use of the Product after the Onset of Adverse Symptoms			
					Continued without Change	Continued with Reduced Amount/Frequency	Stopped	Went to a Hospital
			*n* ^1^	% ^1^	*n*	*n*	*n*	*n*
Experience of adverse events								
Have experienced			75	10.5	22	33	20	1
Symptoms ^2^								
Diarrhea			61	81.3	18	28	15	0
Nausea and vomiting			11	14.7	3	3	5	0
Headache			9	12.0	3	3	3	0
Constipation			5	6.7	1	3	1	1
Anthema and itching			3	4.0	0	2	1	1
Fatigue			5	6.7	0	2	3	0
Loss of appetite			3	4.0	0	2	1	0
Palpitations			2	2.7	1	0	1	0
Worsening of liver function test			1	1.3	0	1	0	0
Worsening of other clinical test values			0					

^1^ Number or percentage of subjects who self-reported “highly probable” and “probable” for causal relationship. ^2^ Multiple answers were allowed. Note: Gastrointestinal symptoms (diarrhea, nausea and vomiting, and constipation) accounted for 92.0% (*n* = 69) of the subjects.
